# Empowerment in the Treatment of Diabetes and Obesity

**DOI:** 10.1155/2016/5671492

**Published:** 2016-12-20

**Authors:** Włodzimierz Łuczyński, Barbara Głowińska-Olszewska, Artur Bossowski

**Affiliations:** Department of Pediatrics, Endocrinology, Diabetology with Cardiology Division, Medical University of Białystok, Waszyngtona 17, 15-274 Białystok, Poland

## Abstract

As the available therapies for diabetes and obesity are not effective enough, diabetologists and educators search for new methods to collaborate with patients in order to support their health behaviors. The aim of this review is to discuss perspectives for the development of new empowerment-type therapies in the treatment of diabetes/obesity. Empowerment is a process whereby patients gain the necessary knowledge to influence their own behavior to improve the quality of their lives. It is carried out in five stages: (1) identify the problem, (2) explain the feelings and meanings, (3) build a plan, (4) act, and (5) experience and assess the execution. Although many years have passed since the advent and popularization of the concept of empowerment, the area remains controversial, mainly with regard to the methodology of therapy. Some previous studies have confirmed the positive effect of empowerment on body weight, metabolic control, and quality of life of patients with type 2 diabetes; however, few studies have been conducted in patients with type 1 diabetes. There is still a need to confirm the effectiveness of empowerment in accordance with Evidence Based Medicine by performing long-term observational studies in a large group of patients. In future, empowerment may become part of the standard of care for patients with diabetes and/or obesity.

## 1. Introduction

Diabetes is one of the most threatening diseases of the 21st century, with epidemiological data indicating that its incidence is increasing [1]. The disease and its treatment are strongly associated with patients' health behavior, as well as their lifestyle and psychosocioeconomic status. Since the available collaboration techniques are not effective enough, new ways to communicate with patients are required in order to support their health behaviors. These new methods should empower patients to take responsibility for their own health and lifestyle. The aim of this article is to discuss the perspectives for the development of empowerment-type therapies, a new approach involving cooperation between the patient with diabetes and/or obesity and health care professionals.

## 2. Treatment of Diabetes Remains a Huge Challenge

In both type 1 and 2 diabetes, taking medication, a healthy lifestyle, diet, and exercise are essential parts of therapy that affect patients' quality of life. Traditionally, the treatment success in patients with diabetes was measured by adherence to medical recommendations. The plan of self-control was adjusted to the patients' illness and not to their priorities, objectives, possibilities, and lifestyle. It is very difficult for patients with diabetes to comply with all medical recommendations and maintain constant normoglycemia [2], even though they are aware of the long-term complications resulting from chronic hyperglycemia. As a result, we often observe continuous stress in both the patient and the health care professionals.

Another issue in diabetes treatment is that implementing complex diabetes therapy requires training of the patient and/or the caregiver. The educational programs currently used to train patients and caregivers in diabetes management are diverse, despite the similar topics and issues [3]. It is also important to note that focusing education programs closely on the medical knowledge and physiology of diabetes alone does not guarantee the correct treatment [4]. Patients must also be willing to make changes to the lifestyle and/or diet and to consistently take any prescribed treatment. What is needed is the unification and standardization of training in diabetes management, including those concerning empowerment and involvement. Such actions should result in more effective teaching and, ultimately, an improvement of metabolic control and decreased long-term complications of diabetes.

## 3. Empowerment Is a New Approach to the Treatment of Diabetes and Obesity

Funnell et al. defined empowerment in 1991 as “a process whereby patients have the knowledge and self-awareness necessary to influence their own behavior and that of others in order to improve the quality of their lives” [5]. This is a change from a paternalistic model to a more equal relationship between the patient and the health care professional. Patient empowerment is a therapeutic technique focused on the patient, in which the patient becomes willing and able to take responsibility for their own life [6]. Empowerment is more than intervention, technique, or strategy; it is a vision to help people to change their behavior and make decisions beneficial to their health. The potential of empowerment is huge; it may change the behavior of not only individuals, but also entire populations and communities. Therefore, empowerment-type therapies can improve the health of entire societies.

The three main pillars of empowerment used in the treatment of diabetes are the belief that (1) diabetes is a patient-managed disease; (2) care for a patient with diabetes should be conducted as education, that is, to provide the necessary knowledge for the patients to make their own decisions; and (3) patients should identify and implement their own treatment goals, which have a real impact on their lives.

The overall aim of empowerment is to provide the patient with critical thinking skills and the ability to make autonomous, informed decisions. Furthermore, empowerment facilitates and supports the patients' reflection on their experience of living with diabetes. Reflection occurring in the doctor-patient relationship (which is characterized by psychological safety, warmth, cooperation, and respect) is essential for independent, positive changes in behavior, emotions, and attitude. In summary, the patients are encouraged to become active participants of self-care, and health care professionals should facilitate this process.

## 4. Methodology of Empowerment Therapy

In recent years, the number of publications highlighting the importance of empowerment as a way to encourage people to take control and responsibility for managing their health has increased. Despite the many articles in the field of empowerment, including in the field of diabetes, there is no agreement from the scientific community concerning the definition, objectives, and methods of conducting this type of therapeutic support [7]. However, in most cases before implementing an empowerment-type approach, the health care professionals must first assess the patient's readiness to change.

### 4.1. Assessment of Readiness to Change: The Use of Diabetes Empowerment Scale

Currently, the readiness of patients with diabetes to change is assessed using the Diabetes Empowerment Scale (DES) and two forms are in use: the full version (28 questions) and the short version (DES-SF, 8 questions) [8]. The DES consists of three parts: (1) managing the psychosocial aspect of diabetes, (2) assessing dissatisfaction and readiness to change, and (3) setting and achieving a diabetes goal. The advantage of this questionnaire is its simple construction, which allows the patients to complete it themselves. In our experience, however, the meanings of questions within the DES form often need to be explained to patients, which may affect the obtained data.

The Diabetes Empowerment Scale (DES) has shown sufficient internal consistency, construct validity, reproducibility, factorial construct validity, and concurrent validity among patients with type 2 diabetes [9]. The results of the DES correlate with age, level of education, disease duration, educational program of diabetes applied, and metabolic control [10, 11]. In a study conducted in a population of Chinese patients with type 2 diabetes, the results of the DES-SF helped predict patients' self-control (assessed according to the Diabetes Self-Care Activities Scale), including diet, physical activity, blood glucose monitoring, medication, taking care of the feet, and metabolic control [12]. The advantage of these studies is the large number of participants. In the BENCH-D study, 2,390 patients with type 2 diabetes were evaluated in terms of the DES-SF [13]. Patients who obtained a result in the top quartile of the DES-SF were younger, more often male, with a higher level of education, better metabolic control, and lower rate of long-term complications compared to patients from other quartiles [13]. Being in the top quartile of the DES-SF also correlated with the results obtained for other surveys, including improved self-control, satisfaction from the treatment of diabetes, and person-centered communication, as well as decreased stress levels. At the same time, significant differences in this regard were noted among the various centers treating diabetes.

The Swedish version of the DES questionnaire showed a correlation between higher diabetes empowerment and longer diabetes duration in adult patients with type 2 diabetes [14]. Women reported a greater need for support than men, and working persons reported greater need for support than retired ones [14]. In addition, persons living with a family had a higher perception of requiring support from relatives than those living alone [14]. The DES has also been used in patients with prediabetes, where it was shown that both self-efficacy and perceptions of the empowerment process determined the health behavior [15]. Interestingly, diabetes empowerment ability (evaluated with DES-FS) and not HbA1c/diabetes complications was a predictor of quality of life among patients with type 2 diabetes in China [16].

In summary, before training and possible reeducation during the years of care occurs, the health care professionals/educators should assess patients' readiness to change with the DES survey.

### 4.2. Principles of Empowerment Therapy

If a patient is willing to change, an empowerment-type therapy should be performed. The stages of therapy are shown as follows.

*Stages of the Empowerment Therapy by Funnell and Anderson [17]*

*Stage I: Identify the Problem*

1. What is the hardest thing about taking care of your diabetes?
2. Tell me about it.
3. Give examples.

*Stage II: Explain Feelings and Meanings*

1. What do you think about this?
2. Why do you think so?
3. How do you think, why is it so?

*Stage III: Build a Plan*

1. What do you want?
2. How could the current situation be changed to make you feel better?
3. Where do you want to be on this after some time (1, 3, 12 months)?
4. What are the options for your conduct?
5. What are the barriers to your goal?
6. Who can help you?
7. What are the costs and benefits of each of your choices?
8. What happens if you do nothing?
9. How important is it to do something with this on a scale from 1 to 10?
10. Build an action plan.

*Stage IV: Action*

1. Do you want to do what is necessary to solve the problem?
2. What are the stages of actions that could be carried out?
3. What do you intend to do?
4. How will you know that you have achieved a success?
5. What will you do right after leaving here?

*Stage V: Experience and Assess the Execution of Your Plan*

1. How did it go?
2. What have you learned?
3. What are the obstacles encountered?
4. What, if anything, would you do differently next time?
5. What will you do after leaving here?

Empowerment allows the patient to gain/regain control over their life; the process helps recognize, promote, and strengthen the capacity of the patients to be responsible for their own lives. This approach recognizes that although health care professionals/educators have expert knowledge of the disease, this does not mean they have expert knowledge of the patient's life. In the course of therapy, the patient becomes a well-informed, active partner in their own care, and the health care professional is given the task of helping the patient in decision-making to achieve the patient's goals and overcome learning barriers.

During empowerment therapy, health care professionals should listen to the patients and ask questions about what the patient needs from them to better manage their diabetes. Health care professionals should stop being responsible for patients and become responsible to them. In addition, health care professionals should show that they care first about the patient as an individual and secondly about their diabetes. The patient, in turn, must understand their role as the person making decisions and take responsibility for them. Furthermore, health care professionals must abandon the illusion that they have control over their patients, including their decisions on glycemic control and effects of therapy. Many health care professionals will be pleased with this type of action, that is, those who define success as creating a relationship with the patient. While developing a relationship with the patient takes more time, this approach increases the efficiency of the visit and may reduce the overall number of visits in the long term. For example, compared to routine consultations, in the Empowerment, Motivation and Medical Adherence (EMMA) program, doctors spoke less and patients more, and modern communication tools were used [18]. This approach resulted in effective education, acceptance by patients, and building a relationship with the therapeutic team [18]. The main difficulty in the EMMA program was the realization of consultations at the scheduled time [18]. It is also feasible to train and graduate peer leaders among adults with diabetes for diabetes self-management support (DSMS) interventions [19]. Therefore, successful empowerment therapy requires a team approach, in which all members must contribute and be willing to change. Lately, it has been shown that psychological skills training for nurses can have a positive impact on patient care including empowerment [20].

Questions about the type of therapy (individual or group), topics, frequency and number of educational visits, the contact time between the educator and the patient, the use of new communication techniques, or ways to tackle barriers to self-control remain unanswered. Furthermore, the review of the literature indicates the effectiveness of empowerment in terms of illness beliefs, compliance to medication, and monitoring blood glucose. There is, however, no long-term observation and evidence of changes in body weight, physical activity, and depression symptoms for large groups of patients with diabetes/obesity undergoing empowerment. Therefore, the process and rules of empowerment should be defined in accordance with the Evidence Based Medicine. This will allow us to carry out reliable, prospective studies to prove the efficacy of this approach and to establish the standards of care for patients with diabetes and/or obesity.

In summary, the fundamental assumptions of empowerment are the following:

1. Patients provide 98% of their own diabetes care.
2. The health and well-being of the patient is the result of their own decisions and actions in daily life.
3. Diabetes is so integrated into the life of the patient that most activities of daily life influence it and, in turn, daily life is influenced by diabetes and its treatment.
4. As patients control their daily decisions, they are responsible for these decisions and the resulting consequences.
5. Patients cannot delegate control or responsibility for the treatment of diabetes, regardless of how much they want to. Even if the doctor is in control, the patient can change his/her mind about any doctor's decision. Thus, the patient controls and is responsible for the treatment.
6. Health care professionals cannot control patients and, therefore, cannot be responsible for the independent decisions of their patients.
7. Health care professionals should do everything in their power to make sure that their patients make informed, independent decisions, that is, that they have the knowledge and understanding of diabetes treatment and an awareness of aspects of their personal lives that affect their decisions.

## 5. The Effects of Empowerment Therapy in Type 2 Diabetes and Obesity

Most studies on the use of empowerment therapy were conducted in adult patients with type 2 diabetes and/or overweight/obesity. The objectives of these studies included better quality of life, normalization of body weight and lipid profile, improved metabolic control, and prevention of diabetic complications. The main limitations of these studies were the small study sizes, short observation times, methodological difficulties in conducting the research and assessment of its effects, and the small differences in treatment effects between the study and reference groups. Therefore, conflicting results of the use of empowerment therapy have been reported.

### 5.1. Effect of Empowerment on Patients' Quality of Life

Empowerment perceptions strongly influence self-efficacy and self-care and thus affect metabolic control in patients with type 2 diabetes [21]. Therefore, to improve patients' health behavior, an empowerment-type therapy should be used instead of an authoritative-type approach. The effects of these therapies are multidirectional. Indeed, participants with type 2 diabetes enrolled in the Patient Empowerment Program were characterized by fewer hospitalizations during the 30-month observation compared to patients who did not participate in the program [22]. In addition, the improvement in the quality of life (through improvement in bodily pain and emotional roles, among others) was noted [23]. Surprisingly, this improvement did not correlate with the number of sessions in which the patient participated [23]. In another intervention, empowerment support through a life coach and pharmacist counselor led to positive effects in terms of self-efficacy, quality of life, and body mass index (BMI), but no improvement in metabolic control (measured by glycated hemoglobin [HbA1c] levels) was observed [24]. Previous studies have confirmed the positive effect of empowerment and education on metabolic control and quality of life of patients with diabetes. However, only a few randomized trials suggest a positive (albeit not very strong) influence of empowerment therapy on mental health-related quality of life independent effects on metabolic control [25]. This may be an additional argument for using this type of patient support.

The so-called readiness to change, measured by a dedicated DES survey, is associated with the quality of life in patients with type 2 diabetes and their treatment. After using the Diabetes Conversation Map™ tool, the proportion of patients with type 2 diabetes showing willingness to start lifestyle changes increased from 20.3% to 65.7% [26]. One of the major problems relating to quality of life is in patients with type 2 diabetes who should have insulin injections introduced at some stage of their treatment. Often the decision is postponed in these patients, as introducing insulin injections is regarded by the patient as a personal failure. The goal of empowerment is, among others, to raise the patient's awareness that this is the next stage of treatment and it leads to better metabolic control [27].

### 5.2. Effect of Empowerment on Patients' Metabolic Control and Lipid Profile

Most studies show a positive effect of empowerment therapy on HbA1c levels and lipid profile. For example, after the 6-month empowerment-type intervention among African-Americans with type 2 diabetes, a statistically significant improvement in metabolic control was observed compared to the control period (in which patients received educational newsletters) [28]. Interestingly, during the control period, there was a statistically significant improvement in blood pressure, cholesterol, diet, and glucose monitoring [28]. Similarly, after the empowerment-type intervention in the Italian SINERGIA program, a particularly strong effect was achieved in patients with type 2 diabetes with very poor metabolic control: that is, the percentage of patients with HbA1c ≥ 9% decreased from 10.5% to 4.3% [29]. Furthermore, in a large group of patients with type 2 diabetes, the use of empowerment therapy not only resulted in improved metabolic control (measured as the percentage of patients with HbA1c ≤ 7.0%) and lipid profile (LDL-cholesterol ≤ 2.6 mmol/L), but also reduced the frequency of doctor's appointments compared to a control (matched) group [30]. The Taiwanese MAGIC study also showed that empowerment therapy was effective in patients with type 2 diabetes; after three months of the intervention in this Taiwanese population, an improvement in metabolic control, self-care behaviors, self-efficacy, and quality of life was observed [31].

Currently, we do not know what specific characteristics determine the success of empowerment therapy. Age, sex, duration of diabetes, and acculturation did not affect the results of empowerment interventions (especially HbA1c results and DES score) among first-generation immigrants from Armenia aged ≥65 years with type 2 diabetes [32]. However, other elements, such as comorbidities, may affect the results. Therefore, there is an ongoing study to examine the use of empowerment in war veterans from southern Texas (USA) with poor metabolic control and accompanying depression, that is, the Healthy Outcomes through Patient Empowerment (HOPE) intervention [33]. This study will examine whether the intervention has positive effects on both diabetes and depression [33].

While most studies conducted to date show positive results, several studies have not confirmed the effectiveness of empowerment in all aspects of diabetes care. For example, in a group of 344 patients with type 2 diabetes, intervention was introduced to assist the patients in their decision-making ability, as well as to provide individually tailored training and more treatment options [34]. After the intervention, there was no statistically significant increase in the empowerment score (determined using a structured questionnaire) and no change in the lipid profile compared to the control group [34]. In another study (the Peer-Led, Empowerment-Based Approach to Self-Management Efforts in Diabetes, or PLEASED study), no improvement in metabolic control in patients with type 2 diabetes was observed after one year of the intervention compared to the diabetes self-management education (DSME) control group [35]. However, there were some positive effects of the intervention in the PLEASED study, which related only to certain risk factors for cardiovascular diseases, such as lipid profile, blood pressure, and BMI [35]. Furthermore, in the group of 569 patients with type 2 diabetes participating in the Community Orientated Diabetes Education (CODE) program, no link between the characteristics of empowerment and metabolic and lipid parameters was found [36]. Frequent contacts through a telephone-based peer support program (PEARL) did not improve cardiometabolic risks or psychological well-being as compared to web-based multicomponent improvement program (JADE) among Chinese patients with type 2 diabetes [37]. The lack of the effectiveness of empowerment in these studies may be due to methodological problems and/or an insufficient number of patients.

### 5.3. Effect of Empowerment on the Long-Term Complications of Diabetes

While we usually focus on a limited number of short-term goals in psychological interventions (including measurements of blood glucose, metabolic control, blood pressure, and lipids), the most important goal is to reduce the long-term complications of diabetes, including cardiovascular disease. When the Patient Empowerment Program (PEP) was used at least once in a large group of patients with type 2 diabetes, a reduction in the occurrence of the first distant vascular complications (including nephropathy) and reduction in overall mortality were observed compared to matched patients without the intervention [38, 39].

### 5.4. Effect on Overweight and Obesity

Overweight and obesity are one of the main problems associated with both type 1 and type 2 diabetes, as they increase the risk of cardiovascular disease in these patients. However, most therapeutic programs used in diabetes are verified based on achieving metabolic control (mainly HbA1c) rather than normalizing body weight. Indeed, only a small number of studies have used the normalization of body weight as a goal. In one of these studies, a positive effect on the normalization of BMI, systolic blood pressure, and stress levels was demonstrated following empowerment therapy among adult patients with type 2 diabetes [40]. In addition, the health self-empowerment workshop that was conducted among 153 low-income adults with overweight/obesity resulted in increased engagement in physical activity and healthy diet, as well as a reduction in BMI and blood pressure, compared to the control group [41].

Very few reports concern the use of empowerment in childhood obesity. The Pediatric Obesity Empowerment Model-Group Medical Visits (POEM-GMV) program examined the use of empowerment in children (average age of 10 years) who were overweight or obese [42]. This study showed that empowerment was effective in terms of reducing the body weight, especially in boys [42]. Moreover, improvement in BMI was accompanied by reduced stress levels, increased physical activity, improved diet, and reduction of watching TV [42].

In summary, while there have been some conflicting reports, overall empowerment therapy seems to improve the quality of life, metabolic control, lipid profile, and BMI and reduce the associated long-term complications (including obesity and cardiovascular disease risk) in patients with type 2 diabetes.

## 6. The Effects of Empowerment Therapy in Type 1 Diabetes

Five basic elements are applied in the treatment of type 1 diabetes: insulin, diet, self-control, exercise, and education. Compliance with medical recommendations in this disease entity is very burdensome for the patient. Effective methods of influencing the emotional burden of type 1 diabetes are unknown. For example, among 2,419 Danish adult patients with type 1 diabetes, high emotional burden occurred more frequently in younger women with comorbidities and chronic abnormal metabolic control [43]. However, the biggest differences in the emotional burden level in this group of patients were caused by low empowerment, poor quality of life, and poor support [43]. In another study, patients with type 1 diabetes living without a partner were characterized by lower DES scores compared with the control group [44]. The DES is also used to assess the effects of support, through the empowerment of parents who are struggling with a new diagnosis of type 1 diabetes in their children [45]. Patients and their caregivers who are notably mentally burdened should be qualified for new therapies that will improve their functioning and quality of life.

The current standard of care in type 1 diabetes is flexible intensive insulin therapy, which should lead to the achievement of correct metabolic balance and good quality of life. However, this system is more complex and technically difficult than other insulin regimens. For example, despite the fact that patients were subjected to empowerment therapy during the Dose Adjustment for Normal Eating (DAFNE) courses, they still expected and required additional support from health care professionals [46]. This support from the health care professionals related to the proper dosage of insulin and encouragement in dealing with problems, among others [46]. In the patients' opinion, they require individual access to support of clinicians. This implies potential limitations on the use of this type of therapy and the need for support from a large therapeutic team.

The German PRIMAS educational program included elements of empowerment for people with type 1 diabetes and resulted in the improvement in both diabetic parameters and the degree of patient satisfaction with their treatment [47]. Similarly, patient-centered communication was associated with empowerment (assessed as greater perceptions of control and competence) in adolescents with type 1 diabetes [48]. Only a few studies have been performed on teenagers and young adults treated with the personal insulin pump, including one utilizing the Guided Self-Determination (GSD) therapeutic program [49]. The recently developed GSD program is based on empowerment and is patient-centered, thereby encouraging supportive problem solving and decision-making skills related to treatment with a personal insulin pump in adolescents with type 1 diabetes [49]. However, it will take time to determine the effects of this program in young people with type 1 diabetes. Another interesting trial MODIAB-Web (person-centered web-based support) was designed to improve self-management in women with type 1 diabetes during pregnancy and early motherhood [50]. Diabetes management in this study was evaluated with the Swedish short version of DES. The results showed that women with HbA1c levels of ≤48 mmL/mol scored higher in the subscales “goal achievement” in SWE-DES scale.

Theoretically all aspects of empowerment including education, self-management, and shared decision-making can be covered by mobile health technologies, so-called mHealth [51]. For example, a mobile-phone-based tool to capture and visualize food intake was effective in empowerment of young people with type 1 diabetes with their diet [52]. However the use of Web portal in the group of young patients with type 1 diabetes and their parents did not show any additional positive influence on the quality of life and empowerment (DES) as compared to the control group [53].

Finally, it is worth considering whether empowerment therapy is better used in patients with long lasting type 1 diabetes rather than at the time of diagnosis. Indeed, empowerment seems to be the ideal tool for obtaining a better quality of life and proper metabolic control in patients who have become tiresome of the disease or who have decreased motivation due to the ongoing routine.

## 7. Difficulties and Barriers in Implementing the Empowerment Treatment

Although many years have passed since the development and popularization of the idea of empowerment, much controversy and misunderstanding in this area remains. The misunderstanding of what empowerment actually entails occurs in both health care professionals and patients. Indeed, our current review highlights that the term empowerment is often used interchangeably, with different intended meanings. For example, some use the term to indicate a simple reeducation process aimed at improving metabolic control and better compliance with medical recommendations; others use the term empowerment to describe a process whereby the patients become motivated and comply with medical recommendations, rather than simply leaving the diabetes care in the patients' hands [39].

The introduction of truly embracing empowerment by health care professionals is extremely difficult because we are profoundly embedded in a traditional approach to care. This is because most educators believe that diabetes education is aimed at increasing patients' adherence with diabetes recommendations. On one hand, these difficulties arise from the type of training of health care professionals. This approach is effective in acute diseases and is focused on convincing the patient to follow the medical recommendations; however, it does not work in chronic diseases, such as diabetes or obesity. The essence of the work of empowerment educators is to provide the information to the patients about the effects and consequences of their health behavior. Health care professionals cannot control the patients and are not responsible for patients' self-control. On the other hand, due to methodological confusion, many educators/health care professionals implement the educational systems, which are based on an incorrect view on empowerment, and therefore the effects of these programs may be confusing [54]. The explanation of empowerment and improved understanding, as well as the standardization of the implementation of the therapy, will benefit both patients and the therapeutic team.

All these limitations can be clearly seen in pregnant women with diabetes: for example, despite talk of empowerment, in practice, professionals often resort to traditional medical models and although women's glycemic control can improve during pregnancy, prolonged improvements in HbA1c patterns are not sustained [55].

Anderson and Funnell indicate a number of misconceptions in the perception of both what empowerment is and what is included in the methods of treatment [54]. It should be emphasized that empowerment does not involve convincing, persuading, or changing patients, or doing something to the patients [54]. The misconceptions of medical staff and misunderstandings on the subject are shown as follows.

*Misconceptions in Empowerment according to Anderson and Funnell: The Beliefs of Health Care Professionals [54]*

1. My patients do not want to be “authorized, empowered” and/or they want me to tell them what to do.
2. I want to use empowerment in my patients to maximize compliance. Empowerment means that the patient is doing everything he should.
3. I'm trying to “authorize, empower” my patients. I am concerned when I fail to get results.
4. There are patients who become “empowered”, patients who do not become “empowered”, and those who cannot be “empowered” because of age, education, and so forth.
5. I'm not sure when to use empowerment. I use empowerment in some patients; this is my spare wheel. I never use empowerment in newly diagnosed patients.
6. Education based on empowerment means focusing only on the problems of the patient.
7. I am using the empowerment approach, because it allows patients to use any diet and adjust their own insulin doses.
8. Responsibility for the health of patients depends entirely on the patients themselves.
9. Empowerment assumes that the patient is able and wants to take responsibility for his diabetes and to be an equal partner in the decision-making process.
10. Empowerment assumes that the doctor simply helps patients to acquire knowledge and skills necessary for informed choices about diabetes.
11. Empowerment assumes that knowledge regarding taking control will be understood and remembered by patients upon communication.

In turn, the barriers in empowerment that are observed in patients are frustration, fatigue, financial problems, transport/access to the therapy center, and scheduling difficulties [56].

Finally, there are a number of general difficulties in implementing empowerment therapy [57]. First, if we assume that the patient to be empowered must have adequate knowledge to make “rational decisions,” we need to explain the term “adequate knowledge.” Furthermore, what is a “rational decision”? What factors (socioeconomic, political, or cultural) determine whether the decision is rational? Obtaining the right knowledge does not necessarily mean a change of behavior will occur. In our daily practice, we observe many patients who have sufficient knowledge to make “rational decisions” but, for unknown reasons, they do not make them. Finally, we must explain in more detail the idea of active partnership. Currently, health care professionals and patients are not certain what the rules and limits of “active partnership” are.

## 8. Conclusions/Summary

Treatment of diabetes, similarly to most chronic diseases, depends on the ability of the patient to cope with everyday problems and to consistently comply with medical recommendations. Therefore, E (for empowerment) has now been added to the classical ABCD (Age, Body Weight, Complications, Duration of the Disease) algorithm in diabetes care [58]. Health care professionals should encourage the patient and improve their ability to make informed decisions concerning managing their own health, as well as improve their self-esteem and responsibility for their own health. Still, there is a need for confirmation of the effectiveness of empowerment therapy in long-term observations on large groups of patients according to the rules of Evidence Based Medicine. In future, empowerment may become part of the standard of care for patients with diabetes and/or obesity.

## References

[1] International Diabetes Federation (2013). *IDF Diabetes Atlas*.

[2] Mane K., Chaluvaraju K. C., Niranjan M. S., Zaranappa T. R., Manjuthej T. R. (2012). Review of insulin and its analogues in diabetes mellitus. *Journal of Basic and Clinical Pharmacy*.

[3] Grant L., Lawton J., Hopkins D. (2013). Type 1 diabetes structured education: what are the core self-management behaviours?. *Diabetic Medicine*.

[4] White R. D. (2012). Patient empowerment and optimal glycemic control. *Current Medical Research and Opinion*.

[5] Funnell M. M., Anderson R. M., Arnold M. S. (1991). Empowerment: an idea whose time has come in diabetes education. *The Diabetes Educator*.

[6] Tol A., Alhani F., Shojaeazadeh D., Sharifirad G., Moazam N. (2015). An empowering approach to promote the quality of life and self-management among type 2 diabetic patients. *Journal of Education and Health Promotion*.

[7] Asimakopoulou K., Newton P., Sinclair A. J., Scambler S. (2012). Health care professionals' understanding and day-to-day practice of patient empowerment in diabetes; time to pause for thought?. *Diabetes Research and Clinical Practice*.

[8] Anderson R. M., Funnell M. M., Fitzgerald J. T., Marrero D. G. (2000). The diabetes empowerment scale: a measure of psychosocial self-efficacy. *Diabetes Care*.

[9] Hara Y., Iwashita S., Okada A. (2014). Development of a novel, short, self-completed questionnaire on empowerment for patients with type 2 diabetes mellitus and an analysis of factors affecting patient empowerment. *BioPsychoSocial Medicine*.

[10] D'Souza M. S. H., Karkada S. N. A., Hanrahan N. P., Venkatesaperumal R., Amirtharaj A. (2015). Do perceptions of empowerment affect glycemic control and self-care among adults with type 2 diabetes?. *Global journal of health science*.

[11] Aveiro M., Santiago L. M., Ferreira P. L., Simões J. A. (2015). Fiability study of diabetes empowerment scale: short version. *Acta Medica Portuguesa*.

[12] Yang S., Hsue C., Lou Q. (2015). Does patient empowerment predict self-care behavior and glycosylated hemoglobin in chinese patients with type 2 diabetes?. *Diabetes Technology & Therapeutics*.

[13] Rossi M. C., Lucisano G., Funnell M. (2015). Interplay among patient empowerment and clinical and person-centered outcomes in type 2 diabetes. The BENCH-D study. *Patient Education and Counseling*.

[14] Isaksson U., Hajdarevic S., Abramsson M., Stenvall J., Hörnsten Å. (2015). Diabetes empowerment and needs for self-management support among people with type 2 diabetes in a rural inland community in northern Sweden. *Scandinavian Journal of Caring Sciences*.

[15] Chen M.-F., Wang R.-H., Hung S.-L. (2015). Predicting health-promoting self-care behaviors in people with pre-diabetes by applying Bandura social learning theory. *Applied Nursing Research*.

[16] Zhu Y., Fish A. F., Li F., Liu L., Lou Q. (2016). Psychosocial factors not metabolic control impact the quality of life among patients with type 2 diabetes in China. *Acta Diabetologica*.

[17] Funnell M. M., Anderson R. M. (2004). Empowerment and self-management of diabetes. *Clinical Diabetes*.

[18] Varming A. R., Hansen U. M., Andrésdóttir G., Husted G. R., Willaing I. (2015). Empowerment, motivation, and medical adherence (EMMA): the feasibility of a program for patient-centered consultations to support medication adherence and blood glucose control in adults with type 2 diabetes. *Patient Preference and Adherence*.

[19] Tang T. S., Funnell M. M., Gillard M., Nwankwo R., Heisler M. (2011). Training peers to provide ongoing diabetes self-management support (DSMS): results from a pilot study. *Patient Education and Counseling*.

[20] Graves H., Garrett C., Amiel S. A., Ismail K., Winkley K. (2016). Psychological skills training to support diabetes self-management: qualitative assessment of nurses’ experiences. *Primary Care Diabetes*.

[21] Lee Y., Shin S., Wang R., Lin K., Lee Y., Wang Y. (2016). Pathways of empowerment perceptions, health literacy, self-efficacy, and self-care behaviors to glycemic control in patients with type 2 diabetes mellitus. *Patient Education and Counseling*.

[22] Wong C. K. H., Wong W. C. W., Wan Y. F., Chan A. K. C., Chan F. W. K., Lam C. L. K. (2016). Effect of a structured diabetes education programme in primary care on hospitalizations and emergency department visits among people with type 2 diabetes mellitus: results from the Patient Empowerment Programme. *Diabetic Medicine*.

[23] Wong C. K. H., Wong W. C. W., Wan E. Y. F., Wong W. H. T., Chan F. W. K., Lam C. L. K. (2015). Increased number of structured diabetes education attendance was not associated with the improvement in patient-reported health-related quality of life: results from patient empowerment programme (PEP). *Health and Quality of Life Outcomes*.

[24] Nishita C., Cardazone G., Uehara D. L., Tom T. (2013). Empowered diabetes management life coaching and pharmacist counseling for employed adults with diabetes. *Health Education and Behavior*.

[25] Sugiyama T., Steers W. N., Wenger N. S., Duru O. K., Mangione C. M. (2015). Effect of a community-based diabetes self-management empowerment program on mental health-related quality of life: a causal mediation analysis from a randomized controlled trial. *BMC Health Services Research*.

[26] Ghafoor E., Riaz M., Eichorst B., Fawwad A., Basit A. (2015). Evaluation of diabetes conversation map™ education tools for diabetes self-management education. *Diabetes Spectrum*.

[27] Spollett G. R. (2012). Insulin initiation in type 2 diabetes: what are the treatment regimen options and how can we best help patients feel empowered?. *Journal of the American Academy of Nurse Practitioners*.

[28] Tang T. S., Funnell M. M., Brown M. B., Kurlander J. E. (2010). Self-management support in “real-world” settings: an empowerment-based intervention. *Patient Education and Counseling*.

[29] Musacchio N., Lovagnini Scher A., Giancaterini A. (2011). Impact of a chronic care model based on patient empowerment on the management of Type2 diabetes: effects of the SINERGIA programme. *Diabetic Medicine*.

[30] Wong C. K. H., Wong W. C. W., Lam C. L. K. (2014). Effects of patient empowerment programme (PEP) on clinical outcomes and health service utilization in type 2 diabetes mellitus in primary care: an observational matched cohort study. *PLoS ONE*.

[31] Chen M.-F., Wang R.-H., Lin K.-C., Hsu H.-Y., Chen S.-W. (2015). Efficacy of an empowerment program for Taiwanese patients with type 2 diabetes: a randomized controlled trial. *Applied Nursing Research*.

[32] Naccashian Z. (2014). The impact of diabetes self-management education on glucose management and empowerment in ethnic Armenians with type 2 diabetes. *Diabetes Educator*.

[33] Cully J. A., Breland J. Y., Robertson S. (2014). Behavioral health coaching for rural veterans with diabetes and depression: a patient randomized effectiveness implementation trial. *BMC Health Services Research*.

[34] Denig P., Schuling J., Haaijer-Ruskamp F., Voorham J. (2014). Effects of a patient oriented decision aid for prioritising treatment goals in diabetes: pragmatic randomised controlled trial. *BMJ*.

[35] Tang T. S., Funnell M. M., Sinco B., Spencer M. S., Heisler M. (2015). Peer-led, empowerment-based approach to self-management efforts in diabetes (PLEASED): a randomized controlled trial in an African American Community. *The Annals of Family Medicine*.

[36] Fitzgerald M., O'Tuathaigh C., Moran J. (2015). Investigation of the relationship between patient empowerment and glycaemic control in patients with type 2 diabetes: a cross-sectional analysis. *BMJ Open*.

[37] Chan J. C. N., Sui Y., Oldenburg B. (2014). Effects of telephone-based peer support in patients with type 2 diabetes mellitus receiving integrated care: a randomized clinical trial. *JAMA Internal Medicine*.

[38] Wong C. K. H., Wong W. C. W., Wan Y. F. (2015). Patient Empowerment Programme (PEP) and risk of microvascular diseases among patients with type 2 diabetes in primary care: a population-based propensity-matched cohort study. *Diabetes Care*.

[39] Wong C. K. H., Wong W. C. W., Wan Y. F. (2015). Patient empowerment programme in primary care reduced all-cause mortality and cardiovascular diseases in patients with type 2 diabetes mellitus: a population-based propensity-matched cohort study. *Diabetes, Obesity and Metabolism*.

[40] Tucker C. M., Lopez M. T., Campbell K. (2014). The effects of a culturally sensitive, empowerment-focused, community-based health promotion program on health outcomes of adults with type 2 diabetes. *Journal of Health Care for the Poor and Underserved*.

[41] Tucker C. M., Butler A., Kaye L. B. (2014). Impact of a culturally sensitive health self-empowerment workshop series on health behaviors/lifestyles, body mass index, and blood pressure of culturally diverse overweight/obese adults. *American Journal of Lifestyle Medicine*.

[42] Geller J. S., Dube E. T., Cruz G. A., Stevens J., Bench K. K. (2015). Pediatric obesity empowerment model group medical visits (POEM-GMV) as treatment for pediatric obesity in an underserved community. *Childhood Obesity*.

[43] Joensen L. E., Almdal T. P., Willaing I. (2016). Associations between patient characteristics, social relations, diabetes management, quality of life, glycaemic control and emotional burden in type 1 diabetes. *Primary Care Diabetes*.

[44] Joensen L. E., Almdal T. P., Willaing I. (2013). Type 1 diabetes and living without a partner: psychological and social aspects, self-management behaviour, and glycaemic control. *Diabetes Research and Clinical Practice*.

[45] Merkel R. M., Wright T. (2012). Parental self-efficacy and online support among parents of children diagnosed with type 1 diabetes mellitus. *Pediatric Nursing*.

[46] Rankin D., Cooke D. D., Elliott J., Heller S. R., Lawton J. (2012). Supporting self-management after attending a structured education programme: a qualitative longitudinal investigation of type 1 diabetes patients' experiences and views. *BMC Public Health*.

[47] Hermanns N., Kulzer B., Ehrmann D., Bergis-Jurgan N., Haak T. (2013). The effect of a diabetes education programme (PRIMAS) for people with type 1 diabetes: results of a randomized trial. *Diabetes Research and Clinical Practice*.

[48] Croom A., Wiebe D. J., Berg C. A. (2011). Adolescent and parent perceptions of patient-centered communication while managing type 1 diabetes. *Journal of Pediatric Psychology*.

[49] Brorsson A. L., Leksell J., Viklund G., Lindholm Olinder A. (2013). A multicentre randomized controlled trial of an empowerment-inspired intervention for adolescents starting continuous subcutaneous insulin infusion—a study protocol. *BMC Pediatrics*.

[50] Linden K., Sparud-Lundin C., Adolfsson A., Berg M. (2016). Well-Being and Diabetes Management in Early Pregnant Women with Type 1 Diabetes Mellitus. *International Journal of Environmental Research and Public Health*.

[51] Krosel M., Svegl L., Vidmar L. (2016). *Mobile Health Technologies—Theories and Applications*.

[52] Froisland D. H., Arsand E. (2015). Integrating visual dietary documentation in mobile-phone-based self-management application for adolescents with type 1 diabetes. *Journal of Diabetes Science and Technology*.

[53] Hanberger L., Ludvigsson J., Nordfeldt S. (2013). Use of a web 2.0 portal to improve education and communication in young patients with families: randomized controlled trial. *Journal of Medical Internet Research*.

[54] Anderson R. M., Funnell M. M. (2010). Patient empowerment: myths and misconceptions. *Patient Education and Counseling*.

[55] Hughes C. (2007). Empowerment: challenges during pregnancy. *Journal of Diabetes Nursing*.

[56] Ramsay Wan C., Vo L., Barnes C. S. (2012). Conceptualizations of patient empowerment among individuals seeking treatment for diabetes mellitus in an urban, public-sector clinic. *Patient Education and Counseling*.

[57] Asimakopoulou K., Gilbert D., Newton P., Scambler S. (2012). Back to basics: re-examining the role of patient empowerment in diabetes. *Patient Education and Counseling*.

[58] Khazrai Y. M., Buzzetti R., Del Prato S., Cahn A., Raz I., Pozzilli P. (2015). The addition of e (Empowerment and Economics) to the ABCD algorithm in diabetes care. *Journal of Diabetes and Its Complications*.

